# Clinical efficacy of sodium bicarbonate in treating pediatric metabolic acidosis with varying level of acid–base balance parameters: a real-world study

**DOI:** 10.1186/s12916-023-03189-8

**Published:** 2023-11-29

**Authors:** Huaqing Liu, Yanmei Cao, Xiaoyan Xue, Zhenjiang Bai, Shuiyan Wu

**Affiliations:** 1 Health Supervision Institute of Gusu District, Suzhou, 215000 Jiangsu China; 2 Department of Occupational Disease Medicine, The Fifth People’s Hospital of Suzhou, The Affiliated Infectious Diseases Hospital of Soochow University, No.10, Guangqian Road, Suzhou, 215131 China; 3 People’s Hospital of Ganzhou, Ganzhou, 341200 Jiangxi China; 4 Pediatric Intensive Care Unit, Children’s Hospital of Soochow University, Suzhou, 215000 Jiangsu China

**Keywords:** Metabolic acidosis, Sodium bicarbonate, Chloride, Mortality, Real-world study

## Abstract

**Background:**

Sodium bicarbonate (SB) infusion is commonly used to correct metabolic acidosis, but its clinical efficacy remains controversial. This study aims to investigate whether acid–base balance parameters should be a consideration for administering SB treatment.

**Methods:**

Children with metabolic acidosis (pH < 7.35 and bicarbonate < 22 mmol/L) who were treated with or without 50 mg/ml SB injection were grouped and extracted from a retrospective cohort database of the Pediatric Intensive Care Unit. The interaction between acid–base balance parameters and SB treatment on mortality was analyzed through mortality curves and cross-effect models. Logistic regression was conducted to estimate the risk of death following SB treatment in the overall children as well as in subgroups, and potential confounding factors were adjusted for. After employing propensity score matching to account for confounding factors, further analysis was performed to evaluate the effectiveness of SB treatment within each chloride subgroup.

**Results:**

A total of 5865 children with metabolic acidosis were enrolled, of which 2462 (42.0%) received SB treatment. In the overall population, it was found that SB treatment did not reduce hospital mortality or 28-day mortality. Interactions between acid–base balance parameters (chloride and anion gap) and SB treatment on mortality were observed. Subgroup analysis clarified that when chloride levels were below 107 mmol/L, children treated with SB had higher in-hospital mortality (29.8% vs 14.9%) and 28-day mortality (26.5% vs 13.4%), with adjusted ORs of 2.065 (95% CI, 1.435–2.97) and 1.947 (95% CI, 1.332–2.846), respectively. In contrast, when chloride levels were greater than or equal to 113 mmol/L, children treated with SB had a shorter stay in the PICU (median: 1.1 days vs 5.1 days, adjusted *p* = 0.004) and lower in-hospital mortality (4.3% vs 10.3%) and 28-day mortality (4.0% vs 8.4%), with adjusted ORs of 0.515 (95% CI, 0.337–0.788) and 0.614 (95% CI, 0.391–0.965), respectively. After controlling for confounding factors through matching, the impact of SB treatment on the risk of death in each chloride subgroup was consistent with the aforementioned results. However, treatment with SB did not significantly increase the risk of death in newborns or children with moderate to severe metabolic acidosis when chloride levels were below 107 mmol/L (*p* > 0.05).

**Conclusions:**

The use of sodium bicarbonate for treating metabolic acidosis has been found to increase mortality in children with low chloride levels but decrease mortality in those with high chloride levels in this study. Further prospective multi-center clinical studies and basic research are needed to validate these findings.

**Supplementary Information:**

The online version contains supplementary material available at 10.1186/s12916-023-03189-8.

## Background

Metabolic acidosis is an acid–base imbalance that depletes the body’s buffers, resulting in a decrease in serum bicarbonate (HCO3^−^) concentration, which leads to a secondary reduction in carbon dioxide (PaCO_2_) levels and a decrease in blood pH [1]. Critically ill patients, such as those with sepsis, severe hypoxemia, and cardiogenic shock, are often susceptible to developing metabolic acidosis, which can cause hemodynamic instability, reduced myocardial contractility, impaired cellular oxygen supply and mitochondrial oxygen consumption, compromised catecholamine reactivity, and an increased risk of mortality [2, 3]. Moderate to severe metabolic acidosis affects around 8.4% of ICU patients, with ICU and hospital mortality rates of 17.3% and 21.5%, respectively [4].

To restore normal cardiovascular function and oxygen delivery to tissues, intravenous sodium bicarbonate (NaHCO_3_, SB) solutions have been commonly administered over the past two decades [5], with the assumption that SB raises extracellular pH by delivering sodium ions and bicarbonate, consequently increasing extracellular strong ion difference (SID) with the sodium ion remaining in its monovalent form. Despite the commonality of this clinical practice, there is inadequate assessment of the effect of SB on biochemical parameters [6], with controversy remaining as to whether treating metabolic acidosis with SB results in improved clinical outcomes [7, 8]. Studies have found that treating adults and children with severe metabolic acidosis with SB does not improve mortality or Sequential Organ Failure Assessment (SOFA) score [9–11]. However, SB treatment has demonstrated the potential to reduce organ damage and 28-day mortality rate in acute kidney injury patients with an Acute Kidney Injury Network (AKIN) score of 2 or 3, reducing the demand for renal replacement therapy [12–14] and facilitating early weaning from mechanical ventilation and shortening ICU stays in hyperlactate sepsis patients [15]. Although it does not improve hemodynamic parameters, some scholars have recommended SB infusion in patients with a pH of less than 7.15 [16, 17]. Nevertheless, this treatment has not been proven to improve overall mortality [9, 18].

Currently, there is no indicator parameter to decide when SB should be administered to patients with metabolic acidosis. Acid–base balance parameters significantly influence pH levels and clinical outcomes, including lactic acid accumulation, reduced chloride (Cl^−^) levels, and disorders in calcium (Ca^2+^) and sodium (Na^+^), which can eventually increase mortality [19–21]. These parameters may potentially impact the effectiveness of SB in increasing extracellular pH, thereby affecting its clinical efficacy. In this study, we analyze real-world data collected from Pediatric Intensive Care Unit (PICU) to investigate the influence of acid–base balance parameters on the clinical outcomes of SB treatment in children with metabolic acidosis.

## Methods

### Objects and data sources

This was a bilingual, open-purpose, single-center, retrospective cohort database developed by the Children’s Hospital at Zhejiang University School of Medicine (http://pic.nbscn.org). This database contains information about children admitted to the PICU, including vital signs, medications, laboratory results, functional balance, diagnostic codes, length of hospital stay, and survival data. The PICU database was constructed using clinical data collected from patients admitted to any of the PICUs between 2010 and 2018. This project received approval from the Institutional Review Board of the Children’s Hospital at Zhejiang University School of Medicine (Hangzhou, China) [22].

### Grouping and definition

Metabolic acidosis was defined as a pH < 7.35 and a bicarbonate (HCO3^−^) level of < 22 mmol/L [23, 24]. Severe metabolic acidosis was defined as pH < 7.20 and HCO3^−^  < 10 mmol/l, and moderate metabolic acidosis was defined as 7.20 ≤ pH < 7.30 and 10 ≤ HCO3^−^  < 19 mmol/l [25]. Children who met the definition of metabolic acidosis during their PICU stay were included in the study, while children who had errors in temporal logic (e.g., discharged earlier than the admission time) and those with missing blood cell, blood biochemistry, and acid–base parameters were excluded. The children were divided into two groups based on whether they received 50 mg/ml SB injection. The dose of SB administered to each child was determined based on their weight and medical condition as assessed by the treating physician. In the treatment group, children who developed metabolic acidosis after the last administration of SB were excluded from the study.

### Primary and secondary outcomes

To screen and clean the data, identification codes including subject, admission, and ICU stay codes were used to eliminate duplicate and ambiguous data. Demographic characteristics, ICD10 diagnosis, in-hospital deaths, blood cell and blood chemistry test parameters detected for the first time after entering the ICU, acid–base balance parameters, and date data were extracted. The following calculations were used to determine age and length of PICU admission: age = PICU admission date − date of birth; and length of PICU stay = PICU discharge date − PICU admission date. Whenever the anion gap (AG) value was missing, the AG value was calculated using the following formula: AG = Na^+^  − Cl^−^  − HCO3^−^.

The primary outcome was in-hospital death, while 28-day death and length of ICU stay were secondary outcomes. A death occurring during hospitalization was classified as an in-hospital death, while deaths occurring within 28 days of PICU admission, including those after discharge from the PICU, were categorized as 28-day death.

### Statistical analysis

Categorical variables are presented as frequencies and percentages. The chi-square test or Fisher exact test was used to compare categorical variables. Measurement variables are presented using the mean and standard deviation or median with inter-quartile range (IQR). While Student’s *t*-test was applied to compare measurement variables between two groups following normal distribution, Wilcoxon two-sample test was used when measurement variables were not complying with normal distribution.

Logistic regression was used to estimate the odds ratio (OR) of death for children treated with SB. To test the stability of the OR, different confounding factors were adjusted in various models, which included demographic characteristics, acid–base balance parameter values, blood cells, blood biochemical parameters, disease diagnosis classification, acidosis severity, sepsis, and surgical treatment. After employing the propensity score matching method, with a clamp value set to less than 0.25, to balance the aforementioned confounding factors between the groups of children receiving SB treatment and those not receiving SB treatment, further analysis was conducted to assess the effectiveness of SB treatment. The comparison of the length of ICU stay between the two groups was adjusted using a generalized linear mixed model (GLMM). Unless otherwise noted, the acid–base balance parameters analyzed in this study were values at the time metabolic acidosis was initially diagnosed.

The acid–base balance parameters were grouped into series and an in-hospital mortality curve was plotted. The locally weighted scatter-plot smoothing (LOWESS) method was used to fit the curve. Subgroup analyses were conducted using cut-off values obtained from the mortality curve. Statistical analysis was performed using SAS 9.4 (SAS Institute, Inc., Cary, NC, USA), and all tests were two-tailed. A *p*-value less than 0.05 was considered statistically significant.

## Results

### General characteristics of children

A total of 5865 children who met the screening criteria were enrolled (Additional file 1: Figure S1). Among the children, 2462 (42.0%) were treated with SB, and the median total dose of SB given was 0.88 g (IQR, 0.5–1.88). Of those treated, 1132 (46.0%) received treatment more than one dosage. Table 1 displays the baseline characteristics of the two groups of children, and Table 2 shows the laboratory parameters for both groups. Additional file 1: Table S1 displays characteristics of children who died and survived from hospital.

**Table 1:** Baseline characteristics of children in two groups

Variables	Without SB, *n* = 3403 (%)	With SB, *n* = 2462 (%)	*p*
Gender			0.883
Female	1433 (42.1)	1032 (41.9)	
Male	1970 (57.9)	1430 (58.1)	
Age (days), median (IQR)	115 (5, 660)	324 (73, 1128)	< 0.001
≤ 28 days	1141 (33.5)	386 (15.7)	< 0.001
28 days–1 year	1098 (32.3)	902 (36.6)	
1–3 years	560 (16.5)	546 (22.2)	
3 years or above	604 (17.7)	628 (25.5)	
The most severe metabolic acidosis			<0.001
Mild	2483 (73.0)	1681 (68.3)	
Moderate	763 (22.4)	665 (27.0)	
Severe	157 (4.6)	116 (4.7)	
Blood/immune diseases	30 (0.9)	93 (3.8)	< 0.001
Circulation system disease	314 (9.2)	220 (8.9)	0.702
Digestive system diseases	376 (11.1)	355 (14.4)	< 0.001
Genitourinary disease	21 (0.6)	238 (9.7)	< 0.001
Congenital diseases	1174 (34.5)	827 (33.6)	0.469
Injury or poisoning	218 (6.4)	178 (7.2)	0.215
Sepsis	96 (2.8)	124 (5.0)	< 0.001
Pneumonia	213 (6.3)	217 (8.8)	< 0.001
Meningoencephalitis	68 (2.0)	80 (3.3)	0.003
Surgery	1716 (50.4)	1300 (52.8)	0.072

**Table 2:** Blood cell, blood biochemistry, and acid–base parameters of children in two groups

Variables	Without SB, *n* = 3403	With SB, *n* = 2462	*p*
pH	7.31 (7.26, 7.33)	7.31 (7.26, 7.33)	0.934
HCO_3_^−^ (mmol/L)	19.70 (17.90, 20.90)	19.30 (17.30, 20.60)	< 0.001
Anion gap	8.10 (4.70, 12.10)	7.10 (3.60, 11.00)	< 0.001
Ca^2+^ (mmol/L)	1.22 (1.11, 1.30)	1.20 (1.10, 1.27)	< 0.001
Cl^−^ (mmol/L)	110.00 (107.00, 113.00)	112.00 (108.00, 115.00)	< 0.001
K^+^ (mmol/L)	3.80 (3.40, 4.40)	3.70 (3.30, 4.30)	< 0.001
Na^+^ (mmol/L)	137.00 (134.00, 140.00)	138.00 (134.00, 141.00)	0.001
Actual base excess (mmol/L)	− 5.80 (− 7.70, − 4.40)	− 6.00 (− 8.20, − 4.60)	< 0.001
Lactate (mmol/L)	2.20 (1.30, 4.10)	2.00 (1.20, 3.90)	< 0.001
PaCO_2_ (mmHg)	40.60 (37.00, 44.70)	39.80 (35.90, 44.10)	< 0.001
Total bilirubin (µmol/L)	13.70 (6.20, 58.00)	8.60 (5.20, 27.60)	< 0.001
Triglyceride (mmol/L)	0.95 (0.57, 1.50)	1.06 (0.68, 1.63)	< 0.001
Total protein (g/L)	56.20 (46.90, 65.20)	59.80 (51.00, 67.10)	< 0.001
Hemoglobin (g/L)	123.00 (108.00, 144.00)	117.00 (102.00, 131.00)	< 0.001
Platelet (10^9^/L)	289.00 (207.00, 379.00)	296.00 (207.00, 387.00)	0.238
Red blood cell (10^12^/L)	4.27 (3.65, 4.73)	4.23 (3.54, 4.68)	0.004
White blood cell (10^9^/L)	10.40 (7.43, 14.86)	9.36 (6.85, 13.11)	< 0.001

Notation: Data are presented as a median with interquartile range (IQR)

### Risk of mortality in overall children treated with SB

There was no statistically significant difference in in-hospital mortality (9.4% VS. 9.8, *p* = 0.616) or 28-day mortality (8.4% VS. 8.4%, *p* = 0.992) between the group treated with SB and the non-treatment group. The adjusted ORs for in-hospital death and 28-day death after SB treatment were 1.023 (95% CI, 0.826–1.269, *p* = 0.833) and 1.096 (95% CI, 0.871–1.378, *p* = 0.435) (Table 3). Further analysis of multiple models, adjusting for confounding factors in different categories, also indicated that SB treatment did not reduce mortality risk in overall children (Additional file 1: Table S2).

**Table 3:** Risk of mortality in overall children treated with sodium bicarbonate

**Endpoint**	**Treatment**	**Death, *n* (%)**		***p***	**Crude**	**Adjusted**^a^	***p***
		**No**	**Yes**		**OR (95% CI)**	**OR (95% CI)**	
Death in hospital	With SB	2230 (90.6)	232 (9.4)	0.616	0.956 (0.802, 1.140)	1.023 (0.826, 1.269)	0.833
	Without SB	3069 (90.2)	334 (9.8)		1	1	
28-day death	With SB	2256 (91.6)	206 (8.4)	0.992	0.999 (0.828, 1.205)	1.096 (0.871, 1.378)	0.435
	Without SB	3118 (91.6)	285 (8.4)		1	1	

Notation: *SB* Sodium bicarbonate, *OR* The odds ratio of death for children treated with SB as compared to those without

^a^a binary logistic multivariate regression model using death as the dependent variable, adjusted for factors including age, sepsis, pneumonia, meningoencephalitis, surgery, disease diagnosis, HCO_3_^−^, Ca^2+^, K^+^, Cl^−^, Na^+^, PaCO_2,_ lactate, total bilirubin, triglyceride, total protein, hemoglobin, red blood cell, white blood cell

### In-hospital mortality of children with SB treatment interlaced with acid–base parameters

**Fig. 1:**
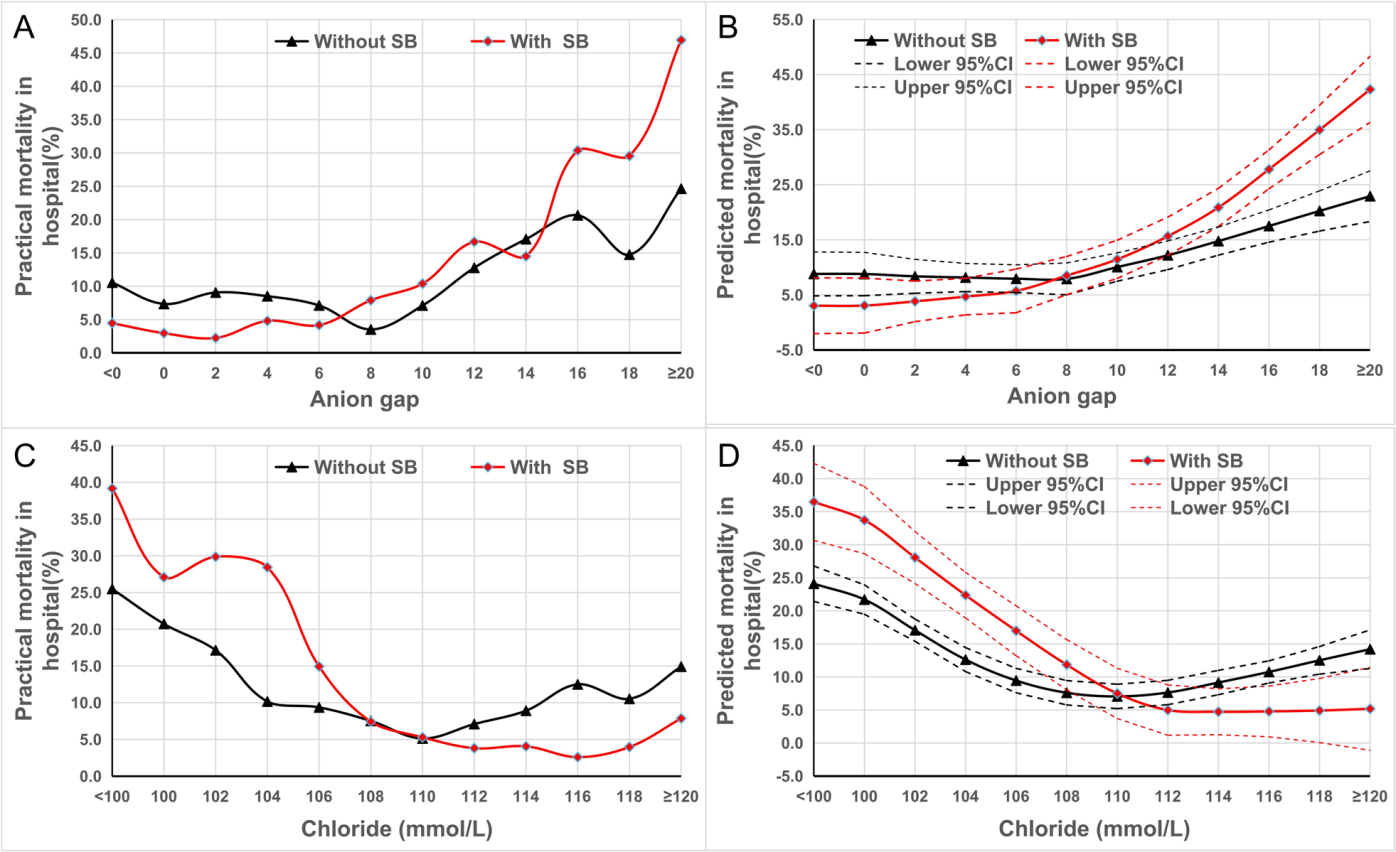
In-hospital mortality curves of children treated with sodium bicarbonate or not, based on varying levels of anion gap and chloride. The ordinate in **A** and **C** represents practical in-hospital mortality, the ordinate in **B** and **D** represents predicted in-hospital mortality using LOWESS, and the abscissa displays anion gap and chloride after grouping into series. SB, sodium bicarbonate

The in-hospital mortality curve indicated a significant difference in the trend of changes between the groups with and without SB treatment after grouping AG or Cl^−^ into series (Fig. 1A, C). When Cl^−^ was low or AG was high, the mortality in the SB treatment group was higher than that in the untreated group. Conversely, when Cl^−^ was high or AG was low, the mortality rate in the SB treatment group was lower than that in the untreated group, indicating that Cl^−^ and AG has an impact on SB treatment. After fitting with LOWESS, the mortality curves of the two groups intersected at Cl^−^ of 110 mmol/L and AG of 8, respectively (Fig. 1B, D). There was no significant difference in the curve between SB treatment and non-treatment after grouping other acid–base balance parameters into series (Additional file 1: Figure S2 and Figure S3).

Further cross-effect analysis also showed a significant cross-effect between Cl^−^, AG, and SB on mortality (Additional file 1: Table S3). After incorporating the interaction effect with Cl^−^ or AG, the impact of SB treatment on mortality shifted from being non-statistically significant to statistically significant.

### Clinical outcomes of SB treatment stratified by chloride level or anion gap

**Fig. 2:**
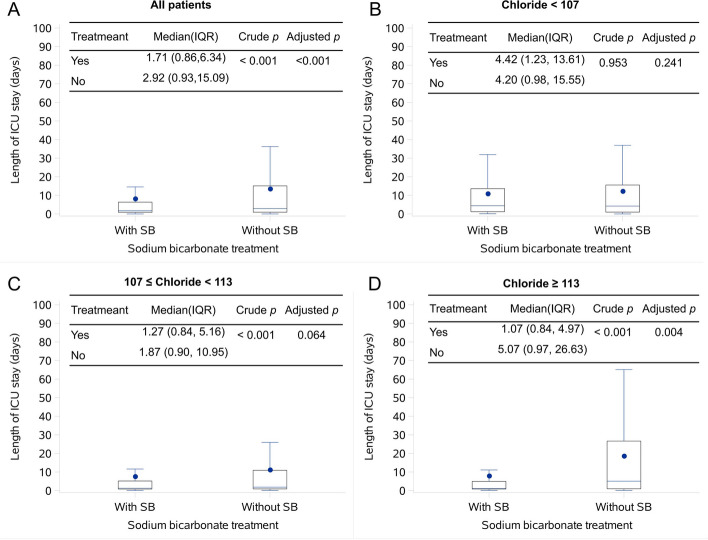
Box-chart of the length of stay in the PICU for surviving children treated with or without sodium bicarbonate. P for comparison on length of PICU stay between two groups. Adjusted *p*, using generalized linear mixed model adjusted factors including age, gender, sepsis, pneumonia, meningoencephalitis, surgery, and grade of metabolic acidosis

When Cl^−^ was lower than 107 mmol/L, children treated with SB had a higher in-hospital mortality (29.8% vs 14.9%, *p* < 0.001) and 28-day mortality (26.5% vs 13.4%, *p* < 0.001), with adjusted ORs of 2.065 (95% CI, 1.435–2.97, *p* < 0.001) and 1.947 (95% CI, 1.332–2.846, *p* = 0.001), respectively (Table 4). Conversely, when Cl^−^ was greater than or equal to 113 mmol/L, children treated with SB had a shorter PICU stay (median: 1.1 days vs 5.1 days, *p* = 0.004, Fig. 2) and lower in-hospital mortality (4.3% vs 10.3%, *p* < 0.001) and 28-day mortality (4.0% vs 8.4%, *p* < 0.001), with adjusted ORs of 0.515 (95% CI, 0.337–0.788, *p* = 0.002) and 0.614 (95% CI, 0.391–0.965, *p* = 0.034), respectively. No statistically significant differences were observed in in-hospital mortality and 28-day mortality between the two groups for Cl^−^ falling between these two values. Further analysis of models correcting for different confounding factors yielded consistent results (Additional file 1: Table S4).

**Table 4:** Risk of mortality in children treated with sodium bicarbonate stratified by chloride level or anion gap

**Subgroups (mmol/L)**	**Endpoint**	**Treatment**	**Death, *n* (%)**		***p***	**Crude**	**Adjusted**^a^	***p***
			**No**	**Yes**		**OR (95% CI)**	**OR (95% CI)**	
Cl^−^ < 107	Death in hospital	With SB	302 (70.2)	128 (29.8)	< 0.001	2.417 (1.824, 3.202)	2.065 (1.435, 2.97)	< 0.001
		Without SB	707 (85.1)	124 (14.9)		1	1	
	28-day death	With SB	316 (73.5)	114 (26.5)	< 0.001	2.34 (1.746, 3.136)	1.947 (1.332, 2.846)	0.001
		Without SB	720 (86.6)	111 (13.4)		1	1	
107 ≤ Cl^−^ < 113	Death in hospital	With SB	929 (94.0)	59 (6.0)	0.354	0.857 (0.619, 1.188)	0.875 (0.595, 1.285)	0.496
		Without SB	1498 (93.1)	111 (6.9)		1	1	
	28-day death	With SB	938 (94.9)	50 (5.1)	0.435	0.869 (0.61, 1.237)	0.958 (0.631, 1.454)	0.841
		Without SB	1516 (94.2)	93 (5.8)		1	1	
Cl^−^ ≥ 113	Death in hospital	With SB	999 (95.7)	45 (4.3)	< 0.001	0.393 (0.273, 0.566)	0.515 (0.337, 0.788)	0.002
		Without SB	864 (89.7)	99 (10.3)		1	1	
	28-day death	With SB	1002 (96.0)	42 (4.0)	< 0.001	0.456 (0.311, 0.67)	0.614 (0.391, 0.965)	0.034
		Without SB	882 (91.6)	81 (8.4)		1	1	
AG < 6	Death in hospital	With SB	968 (96.42)	36 (3.59)	< 0.001	0.385 (0.261, 0.569)	0.475 (0.300, 0.750)	0.001
		Without SB	1046 (91.19)	101 (8.81)		1		
	28-day death	With SB	971 (96.71)	33 (3.29)	< 0.001	0.466 (0.307, 0.706)	0.616 (0.375, 1.012)	0.056
		Without SB	1069 (93.20)	78 (6.80)		1	1	
6 ≤ AG < 12	Death in hospital	With SB	867 (93.03)	65 (6.97)	0.309	1.191 (0.850, 1.670)	1.169 (0.783, 1.747)	0.445
		Without SB	1287 (94.08)	81 (5.92)		1	1	
	28-day death	With SB	880 (94.42)	52 (5.58)	0.684	1.079 (0.747, 1.559)	1.087 (0.702, 1.683)	0.709
		Without SB	1297 (94.81)	71 (5.19)		1	1	
AG ≥ 12	Death in hospital	With SB	395 (5.107)	131 (24.90)	< 0.001	1.606 (1.234, 2.091)	1.400 (1.009, 1.941)	0.044
		Without SB	736 (82.88)	152 (17.12)		1	1	
	28-day death	With SB	405 (77.00)	121 (23.00)	< 0.001	1.652 (1.257, 2.171)	1.480 (1.055, 2.075)	0.023
		Without SB	752 (84.68)	136 (15.32)		1	1	

Notation: *SB* Sodium bicarbonate, *AG* Anion gap, *OR* The odds ratio of death for children treated with SB as compared to those without

^a^a binary logistic multivariate regression model using death as the dependent variable, adjusted for factors including age, sepsis, pneumonia, meningoencephalitis, surgery, disease diagnosis, HCO_3_^−^, Ca^2+^, K^+^, Na^+^, lactate, PaCO_2,_ total bilirubin, triglyceride, total protein, hemoglobin, red blood cell, white blood cell. When calculating the corrected odds ratio (OR) values for each AG subgroup, it did not include HCO3^−^, Na + , and CL but included pH

After stratifying the data according to AG, contrasting outcomes between AG and CL stratification were observed (Table 4). Specifically, SB treatment was found to reduce the risk of death in children with low AG (less than 6) but to increase the risk of death in those with high AG (greater than or equal to 12).

A more detailed subgroup analysis revealed that treatment with SB did not significantly increase or reduce the risk of death in newborns with Cl^−^  < 107 mmol/L (*p* > 0.05) (Additional file 1: Table S5). Additionally, in moderate to severe children with Cl^−^  < 107 mmol/L, SB treatment showed an increase in the in-hospital mortality rate and 28-day mortality by 8.6% and 7.5%, respectively. Nonetheless, the differences were not statistically significant (*p* > 0.05), with adjusted OR = 1.468 (95% CI, 0.933–2.31) and 1.384 (95% CI, 0.869–2.205), respectively (Additional file 1: Table S6). The results of the other subgroups were consistent with those of the subgroups solely divided by Cl^−^ level. Additional file 1: Table S7 presents the characteristics of children with varying levels of chloride.

### Clinical outcomes of SB treatment stratified by chloride level after matching confounding factors

The baseline characteristics of children in each subgroup are presented in Additional file 1: Table S8-10. There were no statistically significant differences in baseline characteristics between SB-treated and non-treated children in each subgroup. After controlling for confounding factors through matching, the impact of SB treatment on the risk of death in each Cl^−^ subgroup was consistent with the aforementioned results. In the subgroup with Cl^−^ lower than 107 mmol/L, children treated with SB had a higher mortality, while in the subgroup with Cl^−^ greater than or equal to 113 mmol/L, children treated with SB had a lower mortality (Table 5).

**Table 5:** Risk of mortality in children treated with sodium bicarbonate stratified by chloride level after matching confounding factors

Subgroups (mmol/L)	Endpoint	Treatment	Death, *n* (%)		*p*	OR (95% CI)
			**No**	**Yes**		
Cl^−^ < 107	Death in hospital	With SB	191 (71.54)	76 (28.46)	0.011	1.685 (1.124, 2.526)
		Without SB	216 (80.90)	51 (19.10)		1
	28-day death	With SB	198 (74.16)	69 (25.84)	0.015	1.674 (1.101, 2.547)
		Without SB	221 (82.77)	46 (17.23)		1
107 ≤ Cl^−^ < 113	Death in hospital	With SB	414 (92.62)	33 (7.38)	1.000	1.000 (0.606, 1.651)
		Without SB	414 (92.62)	33 (7.38)		1
	28-day death	With SB	419 (93.74)	28 (6.26)	0.779	1.082 (0.624, 1.877)
		Without SB	421 (94.18)	26 (5.82)		1
Cl^−^ ≥ 113	Death in hospital	With SB	422 (96.35)	16 ( 3.65)	0.001	0.367(0.203, 0.665)
		Without SB	397 (90.64)	41 ( 9.36)		1
	28-day death	With SB	422 (96.35)	16 ( 3.65)	0.012	0.465 (0.252, 0.858)
		Without SB	405 (92.47)	33 ( 7.53)		1

Notation: *SB* Sodium bicarbonate, *AG* Anion gap, *OR* The odds ratio of death for children treated with SB as compared to those without; confounding factors such as age, sepsis, pneumonia, meningoencephalitis, surgery, disease diagnosis, pH, Cl^−^, Ca^2+^, K^+^, Na^+^, lactate, PaCO_2,_ total bilirubin, triglyceride, total protein, hemoglobin, red blood cell, and white blood cell were matched

## Discussion

This study conducted an exploratory analysis utilizing a real-world Pediatric Intensive Care Unit database and successfully identified that Cl^−^ and AG levels impact the clinical outcomes of SB treatment for metabolic acidosis in children. To the best of our knowledge, this is the first discovery of its kind. This finding has significant directional implications for future research and may even alter clinical treatment strategies.

In previous studies, binary grouping, matching, or local subgroups were commonly utilized for comparative analyses of data [10, 13, 26]. However, these analytical methods are limited because they do not comprehensively explore the data information, making it difficult to identify factors that affect the clinical efficacy of SB. For instance, matching techniques may utilize potential influencing factors as matching variables, rendering their impact effects unobservable. Due to the large sample data, this study was able to group the acid–base balance parameters in a series, which enabled plotting of mortality rates as a function of changes in acid–base balance parameters. The trend of curve changes facilitated visual comparisons between SB-treated and untreated groups.

Notably, the impact of Cl^−^ and AG on the risk of death in the treatment of metabolic acidosis with SB is not limited to a certain point but presents a threshold-dependent bidirectional effect. Considering that AG is an index calculated using chloride, sodium, and carbonate levels, the mortality curve of the two groups only differs after grouping Cl^−^ values. Hence, we can infer that chloride is the fundamental factor that affects the clinical outcome of SB treatment for metabolic acidosis. Specifically, SB increases the risk of death at lower Cl^−^ level, significantly decreases it at higher Cl ^−^ level, and has no effect on the risk of death at intermediate ranges. Further detailed analysis by age groups indicates that when at lower Cl^−^ level, SB treatment does not increase or decrease the risk of neonatal death, which differs from the way SB treatment increases the risk of non-neonatal death. This phenomenon might be due to the fact that neonates have different physiology from that of older children.

In this study, a high proportion of patients with mild metabolic acidosis and a high proportion of patients with mild metabolic acidosis were given SB treatment. However, the clinical efficacy of SB treatment was found to potentially be associated with the patient’s Cl^−^ level, whether it was mild or moderate to severe. In patients with moderate to severe metabolic acidosis at lower Cl^−^ level, the mortality was increased in SB treatment patients. It should be noted that the increased risk of death was not statistically significant, possibly due to the small sample size of this subgroup. A high proportion of patients with mild metabolic acidosis were given SB treatment, and we infer that this is because (1) in China, the guidelines for SB treatment are vague for patients with mild metabolic acidosis, and the concept that SB treatment is only appropriate for moderate to severe patients is not widely recognized; (2) when a patient experiences mild metabolic acidosis, but the condition may continue to deteriorate due to continuous loss of extracellular fluid, doctors may administer small doses of SB treatment as a preventative measure; and (3) the patient’s base excess level is also a factor considered by doctors, as guided by SB China’s medication package insert. For example, in cases of lactic acidemia, mild metabolic acidosis may occur, but if the patient’s base excess is lower than the normal value, then the doctor may choose to give SB treatment.

In the overall sample analysis, our study did not find a significant correlation between SB treatment and the risk of death in patients with metabolic acidosis, which is consistent with the findings of other studies [10–13, 27]. This suggests that the patient samples used in this study are similar to those used in prior studies. Additionally, it supports the rationality of the results observed in this study; namely, the bidirectional effect offsets the increased and reduced risk of death brought about by SB treatment in the overall sample. Among the studies we reviewed, only Fujii et al. reported Cl^−^ values in patients with metabolic acidosis and suggested that SB treatment had no association with mortality [27]. The median value of Cl^−^ in their study falls within the non-correlated interval identified in our study. In another retrospective study of SB treatment in patients with lactic acidosis, the results showed that the administration of sodium bicarbonate was associated with higher mortality rates [28]. Interestingly, we found that the average AG value of patients included in that study was 19.76, which supports our finding that SB treatment increase mortality risk in patients with higher AG levels (corresponding to lower Cl^−^ levels).

There may be several reasons why treatment with SB reduces the risk of death at high levels of chloride. Firstly, hyperchloremic acidosis can cause an increase in the production of nitric oxide, resulting in vasodilation and lowered systemic blood pressure [29]. SB treatment effectively improves patient response to vasoactive drugs, thereby improving hypotension [27, 30]. Secondly, based on Steward’s theory, SB treatment in patients with a strong ion difference increases sodium concentration and normalizes SID, thereby correcting acidosis [31]. Additionally, acute kidney injury is often associated with a chloride load. The improved prognosis observed in the SB group can be attributed to the lower chloride load [32].

Kim HJ et al. found that the follow-up mean lactic acid level was less decreased by at least 10 mg/dL in the lactic acidosis patients who received SB compared to those who did not [28]. The administration of SB was not found to be associated with a shorter time to resolve acidosis in diabetic ketoacidosis (DKA) patients with low Cl^−^ and high AG nor was it found to be associated with a shorter in-hospital length of stay [33, 34]. However, SB treatment can result in a higher incidence of hypokalemia, which requires correction. This could be a contributing factor to the higher mortality rate observed with SB treatment at low Cl^−^ levels. It is important to note that this study was a retrospective single-center study, and as such, there may be systematic biases associated with the single center. Other limitations of this study include the potential preference of doctors for using SB in pediatric patients and the biological mechanism of chloride in this study is unknown. Another possibility that cannot be ruled out is that Cl^−^ may function as an intermediate or accompanying variable. It is plausible that the decisive factor impacts both the efficacy of SB and the level of Cl^−^, or alternatively, it may affect the efficacy of SB by influencing the level of Cl^−^. Consequently, further prospective multi-center clinical studies and basic research are needed to validate these findings.

## Conclusions

The use of sodium bicarbonate for treating metabolic acidosis has been found to increase mortality in children with low chloride levels but decrease mortality in those with high chloride levels in this study. Further prospective multi-center clinical studies and basic research are needed to validate these findings.

### Supplementary Information

**Additional file 1:** Contains materials used throughout the study. **Figure S1.** Screening flow chart. **Table S1.** Comparison of characteristics between children who died in hospital and those who did not die. **Table S2.** Risk of mortality in overall children treated with sodium bicarbonate. **Figure S2.** In-hospital mortality curves of children treated with sodium bicarbonate or not, based on varying levels of pH, bicarbonate, lactate, and partial pressure of carbon dioxide (PCO_2_). **Figure S3.** In-hospital mortality curves of children treated with sodium bicarbonate or not, based on varying levels of actual base excess, ionized calcium, potassium and sodium. **Table S3.** The effect of sodium bicarbonate treatment combined with acid-base parameters on hospital mortality. **Table S4.** Risk of mortality in children treated with sodium bicarbonate stratified by chloride level. **Table S5.** Risk of mortality in children treated with sodium bicarbonate stratified by age and chloride level. **Table S6.** Risk of mortality in children treated with sodium bicarbonate stratified by grade of metabolic acidosis and chloride level. **Table S7.** Comparison of characteristics between children with different levels of chloride. **Table S8.** Baseline characteristics of sodium bicarbonate treated and untreated children matched using propensity score matching method when chloride < 107 mmol/L. **Table S9.** Baseline characteristics of sodium bicarbonate treated and untreated children matched using propensity score matching method when 107 mmol/L≤ chloride < 113 mmol/L. **Table S10.** Baseline characteristics of sodium bicarbonate treated and untreated children matched using propensity score matching method when 113 mmol/L ≤ chloride.

## Data Availability

The datasets used and/or analyzed during the current study are available from the corresponding author on reasonable request.

## Abbreviations

AKIN: Acute Kidney Injury Network
PICU: Pediatric intensive care unit
AG: Anion gap
CI: Confidence interval
DKA: Diabetic ketoacidosis
GLMM: Generalized linear mixed model
IQR: Inter-quartile range
LOWESS: Locally weighted scatter-plot smoothing
OR: Odds ratio
SB: Sodium bicarbonate
SID: Strong ion difference
SOFA: Sequential Organ Failure Assessment
