# Supplementary material for: Clinical efficacy of sodium bicarbonate in treating pediatric metabolic acidosis with varying level of acid–base balance parameters: a real-world study
Source: BMC Med. 2023 Nov 29;21:473. doi: 10.1186/s12916-023-03189-8 (PMC10688456; doi:10.1186/s12916-023-03189-8)
Supplement: Supplementary file 1 — Additional file 1: Contains materials used throughout the study. Figure S1. Screening flow chart. Table S1. Comparison of characteristics between children who died in hospital and those who did not die. Table S2. Risk of mortality in overall children treated with sodium bicarbonate. Figure S2. In-hospital mortality curves of children treated with sodium bicarbonate or not, based on varying levels of pH, bicarbonate, lactate, and partial pressure of carbon dioxide (PCO2). Figure S3. In-hospital mortality curves of children treated with sodium bicarbonate or not, based on varying levels of actual base excess, ionized calcium, potassium and sodium. Table S3. The effect of sodium bicarbonate treatment combined with acid-base parameters on hospital mortality. Table S4. Risk of mortality in children treated with sodium bicarbonate stratified by chloride level. Table S5. Risk of mortality in children treated with sodium bicarbonate stratified by age and chloride level. Table S6. Risk of mortality in children treated with sodium bicarbonate stratified by grade of metabolic acidosis and chloride level. Table S7. Comparison of characteristics between children with different levels of chloride. Table S8. Baseline characteristics of sodium bicarbonate treated and untreated children matched using propensity score matching method when chloride < 107 mmol/L. Table S9. Baseline characteristics of sodium bicarbonate treated and untreated children matched using propensity score matching method when 107 mmol/L≤ chloride < 113 mmol/L. Table S10. Baseline characteristics of sodium bicarbonate treated and untreated children matched using propensity score matching method when 113 mmol/L ≤ chloride. [file 12916_2023_3189_MOESM1_ESM.docx]

**Table S1. Comparison of characteristics between children who died in hospital and those who did not die.**

| **Variables** | **Non-death, n=5299** | **Death, n=566** | ***p*** |
| --- | --- | --- | --- |
| Gender, n(%) |  |  | 0.010 |
| Female | 2256 (42.6) | 209 (36.9) |  |
| Male | 3043 (57.4) | 357 (63.1) |  |
| Age, n(%) |  |  | < 0.001 |
| ≤28 days | 1335 (25.2) | 192 (33.9) |  |
| 28 days -1 year | 1827 (34.5) | 173 (30.6) |  |
| 1-3 year | 1016 (19.2) | 90 (15.9) |  |
| 3 years or above | 1121 (21.1) | 111 (19.6) |  |
| The most severe metabolic acidosis, n(%) |  |  |  |
| Mild | 3936 (74.3) | 228 (40.3) | < 0.001 |
| Moderate | 1220 (23.0) | 208 (36.7) |  |
| Severe | 143 (2.7) | 130 (23.0) |  |
| Blood/immune diseases, n(%) | 97 (1.8) | 26 (4.6) | < 0.001 |
| Circulation system disease, n(%) | 458 (8.6) | 76 (13.4) | < 0.001 |
| Digestive system diseases, n(%) | 699 (13.2) | 32 (5.7) | < 0.001 |
| Genitourinary disease, n(%) | 253 (4.8) | 6 (1.1) | < 0.001 |
| Congenital diseases, n(%) | 1904 (35.9) | 97 (17.1) | < 0.001 |
| Mental disorders, n(%) | 291 (5.5) | 40 (7.1) | 0.123 |
| Musculoskeletal and hoof disorders, n(%) | 278 (5.3) | 36 (6.4) | 0.263 |
| Perinatal disorders, n(%) | 1101 (20.8) | 126 (22.3) | 0.409 |
| Tumor, n(%) | 240 (4.5) | 31 (5.5) | 0.307 |
| Injury or poisoning, n(%) | 321 (6.1) | 75 (13.3) | < 0.001 |
| Unclassifiable disorders, n(%) | 581 (11.0) | 58 (10.3) | 0.603 |
| Sepsis, n(%) | 175 (3.3) | 45 (8.0) | < 0.001 |
| Pneumonia, n(%) | 354 (6.7) | 76 (13.4) | < 0.001 |
| Meningoencephalitis, n(%) | 116 (2.2) | 32 (5.7) | < 0.001 |
| Surgery, n(%) | 2935 (55.4) | 81 (14.3) | < 0.001 |
| Total bilirubin (umol/L), median (IQR) | 10.20 (5.70, 47.30) | 17.50 (6.50, 63.30) | < 0.001 |
| Triglyceride (mmol/L), median (IQR) | 1.00 (0.62, 1.54) | 0.98 (0.59, 1.72) | 0.740 |
| Total protein (g/L), median (IQR) | 58.7 (49.5, 66.6) | 51.1 (41.8, 59.1) | < 0.001 |
| Hemoglobin (g/L), median (IQR) | 121.0 (106.0, 137.0) | 114.5 (94.0, 140.0) | < 0.001 |
| Platelet (10^9^/L), median (IQR) | 298.0 (218.0, 387.0) | 201.5 (110.0, 312.0) | < 0.001 |
| Red blood cell (10^12^/L), median (IQR) | 4.29 (3.67, 4.72) | 3.87 (3.08, 4.56) | < 0.001 |
| White blood cell (10^9^/L), median (IQR) | 9.86 (7.17, 13.79) | 11.26 (6.75, 17.17) | 0.004 |

**Table S2. Risk of mortality in overall children treated with sodium bicarbonate.**

| **Endpoint** | Model ^#^ |  | **Adjusted OR (95%CI)** | ***p*** |
| --- | --- | --- | --- | --- |
| Death in hospital | Model 1 ^@^ |  | 0.967 (0.788, 1.188) | 0.751 |
|  | Model 2 * |  | 0.939 (0.758, 1.161) | 0.560 |
| 28-day Death | Model 1 ^@^ |  | 1.035 (0.834, 1.285) | 0.754 |
|  | Model 2 * |  | 1.096 (0.871, 1.378) | 0.435 |

Notation: OR, the odds ratio of death for children treated with SB as compared to those without; # A binary logistic multivariate regression model using death as the dependent variable; @ Model 1, adjusted for factors including age, gender, sepsis, pneumonia, meningoencephalitis, surgery, disease diagnosis and grade of metabolic acidosis; * Model 2, adjusted for factors including age, gender, sepsis, pneumonia, meningoencephalitis, surgery, pH, Ca^2+^, K^+^, Cl^-^, Na^+^, PCO_2,_ lactate, total bilirubin, triglyceride, total protein, hemoglobin, red blood cell, white blood cell and platelet.

**Table S3. The effect of sodium bicarbonate treatment combined with acid-base parameters on hospital mortality.**

| Without cross effect ^#^ | | | |  | With cross effect ^#^ | | | |
| --- | --- | --- | --- | --- | --- | --- | --- | --- |
| Independent variables | DF | Wald chi-square | *p* |  | Independent variables | DF | Wald chi-square | *p* |
| Anion gap | 1 | 177.4 | < 0.001 |  | Anion gap | 1 | 204.3 | < 0.001 |
| SB treatment | 1 | 0.083 | 0.773 |  | SB treatment | 1 | **32.6** | **< 0.001** |
|  |  |  |  |  | Anion gap × SB treatment | 1 | **49.63** | **< 0.001** |
| Cl^-^ | 1 | 81.35 | < 0.001 |  | Cl^-^ | 1 | 84.18 | < 0.001 |
| SB treatment | 1 | 0.093 | 0.761 |  | SB treatment | 1 | **34.11** | **< 0.001** |
|  |  |  |  |  | Cl^-^ × SB treatment | 1 | **34.01** | **< 0.001** |
| HCO_3_^-^ | 1 | 224.6 | < 0.001 |  | HCO_3_^-^ | 1 | 224.1 | < 0.001 |
| SB treatment | 1 | 1.725 | 0.189 |  | SB treatment | 1 | 0.82 | 0.365 |
|  |  |  |  |  | HCO_3_^-^ **×** SB treatment | 1 | 1.562 | 0.211 |
| Ca^2+^ | 1 | 24.61 | < 0.001 |  | Ca^2+^ | 1 | 28.23 | < 0.001 |
| SB treatment | 1 | 0.78 | 0.377 |  | SB treatment | 1 | 3.226 | 0.072 |
|  |  |  |  |  | Ca^2+^ **×** SB treatment | 1 | 3.808 | 0.051 |
| Na^+^ | 1 | 5.405 | 0.020 |  | Na^+^ | 1 | 6.037 | 0.014 |
| SB treatment | 1 | 0.205 | 0.651 |  | SB treatment | 1 | 1.446 | 0.229 |
|  |  |  |  |  | Na^+^ **×** SB treatment | 1 | 1.504 | 0.22 |
| Lactate | 1 | 412.7 | < 0.001 |  | Lactate | 1 | 406.7 | < 0.001 |
| SB treatment | 1 | 0.036 | 0.850 |  | SB treatment | 1 | 1.848 | 0.174 |
|  |  |  |  |  | Lactate **×** SB treatment | 1 | 4.164 | 0.041 |
| PaCO_2_ | 1 | 113 | < 0.001 |  | PaCO_2_ | 1 | 108.8 | < 0.001 |
| SB treatment | 1 | 0.000 | 0.998 |  | SB treatment | 1 | 0.108 | 0.742 |
|  |  |  |  |  | PaCO_2_ **×** SB treatment | 1 | 0.118 | 0.732 |
| Actual base excess | 1 | 354.6 | < 0.001 |  | Actual base excess | 1 | 345.8 | < 0.001 |
| SB treatment | 1 | 0.958 | 0.328 |  | SB treatment | 1 | 1.644 | 0.200 |
|  |  |  |  |  | Actual base excess **×** SB treatment | 1 | 0.833 | 0.361 |
| pH | 1 | 362.8 | < 0.001 |  | pH | 1 | 352.7 | < 0.001 |
| SB treatment | 1 | 0.149 | 0.700 |  | SB treatment | 1 | 0.957 | 0.328 |
|  |  |  |  |  | pH **×** SB treatment | 1 | 0.97 | 0.325 |

Notation: # a binary logistic multivariate regression model using death as the dependent variable. DF, degree of freedom. SB, sodium bicarbonate.

**Table S4. Risk of mortality in children treated with sodium bicarbonate stratified by chloride level.**

| **Subgroups (mmol/L)** | **Endpoint** | Model ^#^ |  | **Adjusted OR (95%CI)** | ***p*** |
| --- | --- | --- | --- | --- | --- |
| Cl^-^ < 107 | Death in hospital | Model 1 ^@^ |  | 1.931 (1.382, 2.698) | < 0.001 |
|  |  | Model 2 * |  | 1.956 (1.359, 2.814) | < 0.001 |
|  | 28-day Death | Model 1 ^@^ |  | 1.847 (1.306, 2.610) | 0.001 |
|  |  | Model 2 * |  | 1.841 (1.260, 2.690) | 0.002 |
| 107 ≤ Cl^-^ < 113 | Death in hospital | Model 1 ^@^ |  | 0.885 (0.603, 1.300) | 0.535 |
|  |  | Model 2 * |  | 0.786 (0.536, 1.152) | 0.217 |
|  | 28-day Death | Model 1 ^@^ |  | 0.991 (0.657, 1.493) | 0.964 |
|  |  | Model 2 * |  | 0.831 (0.547, 1.262) | 0.386 |
| Cl^-^ ≥ 113 | Death in hospital | Model 1 ^@^ |  | 0.454 (0.300, 0.687) | < 0.001 |
|  |  | Model 2 * |  | 0.463 (0.304, 0.706) | < 0.001 |
|  | 28-day Death | Model 1 ^@^ |  | 0.538 (0.348, 0.832) | 0.005 |
|  |  | Model 2 * |  | 0.551 (0.352, 0.862) | 0.009 |

Notation: OR, the odds ratio of death for children treated with SB as compared to those without; # A binary logistic multivariate regression model using death as the dependent variable; @ Model 1, adjusted for factors including age, gender, sepsis, pneumonia, meningoencephalitis, surgery, disease diagnosis and grade of metabolic acidosis; * Model 2, adjusted for factors including age, gender, sepsis, pneumonia, meningoencephalitis, surgery, pH, Ca2+, K+, Na+, PCO2, lactate, total bilirubin, triglyceride, total protein, hemoglobin, red blood cell, white blood cell and platelet.

**Table S5. Risk of mortality in children treated with sodium bicarbonate stratified by age and chloride level.**

| **Age (days)** | **Chloride (mmol/L)** | **Endpoint** | **Treatment** | **Death, n (%)** | | ***p*** |  | **Crude** |  | **Adjusted#** | ***p*** |
| --- | --- | --- | --- | --- | --- | --- | --- | --- | --- | --- | --- |
|  |  |  |  | **No** | **Yes** |  |  | **OR (95%CI)** |  | **OR (95%CI)** |  |
| Age ≤ 28 | Cl^-^ < 107 | Death in hospital | With SB | 43 (75.4) | 14 (24.6) | 0.699 |  | 1.143 (0.581, 2.246) |  | 1.242 (0.558, 2.764) | 0.596 |
|  |  |  | Without SB | 186 (77.8) | 53 (22.2) |  |  | 1 |  | 1 |  |
|  |  | 28-day Death | With SB | 46 (80.7) | 11 (19.3) | 0.894 |  | 0.952 (0.459, 1.974) |  | 0.962 (0.406, 2.282) | 0.930 |
|  |  |  | Without SB | 191 (79.9) | 48 (20.1) |  |  |  |  |  |  |
|  | 107 ≤ Cl^-^ < 113 | Death in hospital | With SB | 149 (92.0) | 13 (8.0) | 0.148 |  | 0.630 (0.336, 1.183) |  | 0.589 (0.290, 1.196) | 0.143 |
|  |  |  | Without SB | 419 (87.8) | 58 (12.2) |  |  | 1 |  | 1 |  |
|  |  | 28-day Death | With SB | 149 (92.0) | 13 (8.0) | 0.329 |  | 0.729 (0.386, 1.378) |  | 0.708 (0.346, 1.451) | 0.346 |
|  |  |  | Without SB | 426 (89.3) | 51 (10.7) |  |  | 1 |  | 1 |  |
|  | Cl^-^ ≥ 113 | Death in hospital | With SB | 160 (95.8) | 7 (4.2) | 0.009 |  | 0.352 (0.156, 0.795) |  | 0.333 (0.136, 0.818) | 0.017 |
|  |  |  | Without SB | 378 (88.9) | 47 (11.1) |  |  | 1 |  | 1 |  |
|  |  | 28-day Death | With SB | 161 (96.4) | 6 (3.6) | 0.031 |  | 0.391 (0.162, 0.944) |  | 0.378 (0.144, 0.997) | 0.049 |
|  |  |  | Without SB | 388 (91.3) | 37 (8.7) |  |  | 1 |  | 1 |  |
| Age > 28 | Cl^-^ < 107 | Death in hospital | With SB | 259 (69.4) | 114 (30.6) | < 0.001 |  | 3.230 (2.318, 4.500) |  | 1.910 (1.322, 2.760) | 0.001 |
|  |  |  | Without SB | 521 (88.0) | 71 (12.0) |  |  | 1 |  | 1 |  |
|  |  | 28-day Death | With SB | 270 (72.4) | 103 (27.6) | < 0.001 |  | 3.203 (2.266, 4.528) |  | 1.914 (1.309, 2.800) | 0.001 |
|  |  |  | Without SB | 529 (89.4) | 63 (10.6) |  |  | 1 |  | 1 |  |
|  | 107 ≤ Cl^-^ < 113 | Death in hospital | With SB | 780 (94.4) | 46 (5.6) | 0.376 |  | 1.201 (0.800, 1.801) |  | 0.763 (0.484, 1.203) | 0.244 |
|  |  |  | Without SB | 1079 (95.3) | 53 (4.7) |  |  | 1 |  | 1 |  |
|  |  | 28-day Death | With SB | 789 (95.5) | 37 (4.5) | 0.393 |  | 1.217 (0.775, 1.911) |  | 0.801 (0.487, 1.316) | 0.381 |
|  |  |  | Without SB | 1090 (96.3) | 42 (3.7) |  |  | 1 |  | 1 |  |
|  | Cl^-^ ≥ 113 | Death in hospital | With SB | 839 (95.7) | 38 (4.33) | < 0.001 |  | 0.423 (0.275, 0.653) |  | 0.380 (0.24, 0.602) | < 0.001 |
|  |  |  | Without SB | 486 (90.3) | 52 (9.7) |  |  | 1 |  | 1 |  |
|  |  | 28-day Death | With SB | 841 (95.9) | 36 (4.1) | 0.001 |  | 0.481 (0.305, 0.757) |  | 0.444 (0.275, 0.719) | 0.001 |
|  |  |  | Without SB | 494 (91.8) | 44 (8.2) |  |  | 1 |  | 1 |  |

Notation: SB, sodium bicarbonate; OR, the odds ratio of death for children treated with SB as compared to those without; # A binary logistic multivariate regression model using death as the dependent variable, adjusted for factors including gender, sepsis, pneumonia, meningoencephalitis, surgery and grade of metabolic acidosis.

**Table S6. Risk of mortality in children treated with sodium bicarbonate stratified by grade of metabolic acidosis and chloride level.**

| **Metabolic acidosis** | **Chloride (mmol/L)** | **Endpoint** | **Treatment** | **Death, n (%)** | | ***p*** |  | **Crude** |  | **Adjusted#** | ***p*** |
| --- | --- | --- | --- | --- | --- | --- | --- | --- | --- | --- | --- |
|  |  |  |  | **No** | **Yes** |  |  | **OR (95%CI)** |  | **OR (95%CI)** |  |
| Mild | Cl^-^ < 107 | Death in hospital | With SB | 190 (76.6) | 58 (23.4) | <0.001 |  | 3.308 (2.185, 5.009) |  | 2.269 (1.427, 3.607) | 0.001 |
|  |  |  | Without SB | 531 (91.5) | 49 (8.5) |  |  | 1 |  | 1 |  |
|  |  | 28-day Death | With SB | 197 (79.4) | 51 (20.6) | <0.001 |  | 3.233 (2.088, 5.007) |  | 2.223 (1.367, 3.614) | 0.001 |
|  |  |  | Without SB | 537 (92.6) | 43 (7.4) |  |  | 1 |  | 1 |  |
|  | 107 ≤ Cl^-^ < 113 | Death in hospital | With SB | 710 (97.4) | 19 (2.6) | 0.292 |  | 0.746 (0.431, 1.29) |  | 0.741 (0.415, 1.324) | 0.312 |
|  |  |  | Without SB | 1198 (96.5) | 43 (3.5) |  |  | 1 |  | 1 |  |
|  |  | 28-day Death | With SB | 715 (98.1) | 14 (1.9) | 0.106 |  | 0.603 (0.325, 1.119) |  | 0.606 (0.316, 1.163) | 0.132 |
|  |  |  | Without SB | 1202 (96.9) | 39 (3.1) |  |  | 1 |  | 1 |  |
|  | Cl^-^ ≥ 113 | Death in hospital | With SB | 683 (97.0) | 21 (3.0) | 0.012 |  | 0.505 (0.293, 0.87) |  | 0.53 (0.292, 0.963) | 0.037 |
|  |  |  | Without SB | 624 (94.3) | 38 (5.7) |  |  | 1 |  | 1 |  |
|  |  | 28-day Death | With SB | 685 (97.3) | 19 (2.7) | 0.027 |  | 0.529 (0.298, 0.939) |  | 0.588 (0.314, 1.101) | 0.097 |
|  |  |  | Without SB | 629 (95.0) | 33 (5.0) |  |  | 1 |  | 1 |  |
| Moderate or Severe | Cl^-^ < 107 | Death in hospital | With SB | 112 (61.5) | 70 (38.5) | 0.062 |  | 1.467 (0.98, 2.194) |  | 1.468 (0.933, 2.31) | 0.097 |
|  |  |  | Without SB | 176 (70.1) | 75 (29.9) |  |  | 1 |  | 1 |  |
|  |  | 28-day Death | With SB | 119 (65.4) | 63 (34.6) | 0.093 |  | 1.425 (0.943, 2.154) |  | 1.384 (0.869, 2.205) | 0.171 |
|  |  |  | Without SB | 183 (72.9) | 68 (27.1) |  |  | 1 |  | 1 |  |
|  | 107 ≤ Cl^-^ < 113 | Death in hospital | With SB | 219 (84.6) | 40 (15.4) | 0.322 |  | 0.806 (0.525, 1.236) |  | 0.845 (0.527, 1.355) | 0.485 |
|  |  |  | Without SB | 300 (81.5) | 68 (18.5) |  |  | 1 |  | 1 |  |
|  |  | 28-day Death | With SB | 223 (86.1) | 36 (13.9) | 0.785 |  | 0.939 (0.595, 1.48) |  | 1.083 (0.656, 1.788) | 0.755 |
|  |  |  | Without SB | 314 (85.3) | 54 (14.7) |  |  | 1 |  | 1 |  |
|  | Cl^-^ ≥ 113 | Death in hospital | With SB | 316 (92.9) | 24 (7.1) | <0.001 |  | 0.299 (0.181, 0.493) |  | 0.264 (0.152, 0.46) | < 0.001 |
|  |  |  | Without SB | 240 (79.7) | 61 (20.3) |  |  | 1 |  | 1 |  |
|  |  | 28-day Death | With SB | 317 (93.2) | 23 (6.8) | <0.001 |  | 0.382 (0.227, 0.646) |  | 0.324 (0.181, 0.581) | < 0.001 |
|  |  |  | Without SB | 253 (84.0) | 48 (16.0) |  |  | 1 |  | 1 |  |

Notation: SB, sodium bicarbonate; OR, the odds ratio of death for children treated with SB as compared to those without; # A binary logistic multivariate regression model using death as the dependent variable, adjusted for factors including age, gender, sepsis, pneumonia, meningoencephalitis and surgery.

**Table S7. Comparison of characteristics between children with different levels of chloride.**

| **Variables** | **Cl^-^ < 107,**  **n=1261** | **107 ≤ Cl^-^ < 113, n=2597** | **Cl^-^ ≥ 113,**  **N=2007** | ***P*** |
| --- | --- | --- | --- | --- |
| Gender, n(%) |  |  |  | 0.180 |
| Female | 507 (40.2) | 1086 (41.8) | 872 (43.5) |  |
| Male | 754 (59.8) | 1511 (58.2) | 1135 (56.5) |  |
| Age, n(%) |  |  |  | < 0.001 |
| ≤28 days | 296 (23.5) | 639 (24.6) | 592 (29.5) |  |
| 28 days -1 year | 444 (35.2) | 882 (34.0) | 674 (33.6) |  |
| 1-3 year | 208 (16.5) | 487 (18.7) | 411 (20.5) |  |
| 3 years or above | 313 (24.8) | 589 (22.7) | 330 (16.4) |  |
| The most severe metabolic acidosis, n(%) |  |  |  | < 0.001 |
| Mild | 828 (65.7) | 1970 (75.9) | 1366 (68.1) |  |
| Moderate | 325 (25.8) | 542 (20.9) | 561 (27.9) |  |
| Severe | 108 (8.6) | 85 (3.3) | 80 (4.0) |  |
| Blood/immune diseases, n(%) | 48 (3.8) | 42 (1.6) | 33 (1.6) | < 0.001 |
| Circulation system disease, n(%) | 120 (9.5) | 235 (9.1) | 179 (8.9) | 0.839 |
| Digestive system diseases, n(%) | 132 (10.5) | 342 (13.2) | 257 (12.8) | 0.050 |
| Genitourinary disease, n(%) | 29 (2.3) | 102 (3.9) | 128 (6.4) | < 0.001 |
| Congenital diseases, n(%) | 333 (26.4) | 970 (37.3) | 698 (34.8) | < 0.001 |
| Mental disorders, n(%) | 90 (7.1) | 117 (4.5) | 124 (6.2) | 0.002 |
| Musculoskeletal and hoof disorders, n(%) | 86 (6.8) | 139 (5.3) | 89 (4.4) | 0.013 |
| Perinatal disorders, n(%) | 236 (18.7) | 514 (19.8) | 477 (23.8) | < 0.001 |
| Tumor, n(%) | 99 (7.9) | 187 (7.2) | 127 (6.3) | 0.232 |
| Injury or poisoning, n(%) | 118 (9.4) | 173 (6.7) | 105 (5.2) | < 0.001 |
| Unclassifiable disorders, n(%) | 168 (13.3) | 248 (9.5) | 223 (11.1) | 0.002 |
| Sepsis, n(%) | 81 (6.4) | 73 (2.8) | 66 (3.3) | < 0.001 |
| Pneumonia, n(%) | 145 (11.5) | 175 (6.7) | 110 (5.5) | < 0.001 |
| Meningoencephalitis, n(%) | 51 (4.0) | 45 (1.7) | 52 (2.6) | < 0.001 |
| Surgery, n(%) | 488 (38.7) | 1467 (56.5) | 1061 (52.9) | < 0.001 |
| Total bilirubin (umol/L), median (IQR) | 12.80 (6.10,48.50) | 10.10 (5.70,44.70) | 9.80 (5.50,53.40) | 0.002 |
| Triglyceride (mmol/L), median (IQR) | 1.02 (0.64,1.65) | 0.99 (0.63,1.50) | 0.99 (0.59,1.55) | 0.154 |
| Total protein (g/L), median (IQR) | 55.7 (46.5, 64.7) | 59.2 (50.1, 67.0) | 57.5 (48.2, 65.7) | < 0.001 |
| Hemoglobin (g/L), median (IQR) | 116 (100, 132) | 121 (107, 136) | 122 (107, 142) | < 0.001 |
| Platelet (10^9^/L), median (IQR) | 275.0 (178.0,375.0) | 299 (220, 387) | 290.0 (207, 380) | < 0.001 |
| Red blood cell (10^12^/L), median (IQR) | 4.02 (3.34, 4.58) | 4.29 (3.67, 4.72) | 4.32 (3.72, 4.77) | < 0.001 |
| White blood cell (10^9^/L), median (IQR) | 10.58 (7.06, 15.36) | 9.87 (7.17, 13.82) | 9.79 (7.19, 13.62) | 0.059 |

**Table S8. Baseline characteristics of sodium bicarbonate treated and untreated children matched using propensity score matching method when chloride < 107 mmol/L.**

| **Variables** | **Without SB, n=267** | **With SB, n=267** | ***p*** |
| --- | --- | --- | --- |
| Gender, n(%) |  |  | 0.599 |
| Female | 115 (43.07) | 109 (40.82) |  |
| Male | 152 (56.93) | 158 (59.18) |  |
| Age, n(%) |  |  | 0.500 |
| ≤28 days | 50 (18.73) | 38 (14.23) |  |
| 28 days -1 year | 100 (37.45) | 103 (38.58) |  |
| 1-3 year | 43 (16.10) | 51 (19.10) |  |
| 3 years or above | 74 (27.72) | 75 (28.09) |  |
| Metabolic acidosis grade, n(%) |  |  |  |
| Mild | 217 (81.27) | 224 (83.90) | 0.379 |
| Moderate | 39 (14.61) | 29 (10.86) |  |
| Severe | 11 (4.12) | 14 (5.24) |  |
| Blood/immune diseases, n(%) | 11 (4.12) | 13 (4.87) | 0.676 |
| Circulation system disease, n(%) | 27 (10.11) | 29 (10.86) | 0.778 |
| Digestive system diseases, n(%) | 27 (10.11) | 33 (12.36) | 0.411 |
| Genitourinary disease, n(%) | 7 (2.62) | 9 (3.37) | 0.612 |
| Congenital diseases, n(%) | 51 (19.10) | 52 (19.48) | 0.913 |
| Injury or poisoning, n(%) | 32 (11.99) | 30 (11.24) | 0.787 |
| Sepsis, n(%) | 25 (9.36) | 21 (7.87) | 0.537 |
| Pneumonia, n(%) | 43 (16.10) | 43 (16.10) | 1.000 |
| Meningoencephalitis, n(%) | 17 (6.37) | 16 (5.99) | 0.857 |
| Surgery, n(%) | 71 (26.59) | 74 (27.72) | 0.770 |
| pH, median (IQR) | 7.29 (7.21,7.33) | 7.30 (7.18,7.33) | 0.928 |
| Ca^2+^ (mmol/L), median (IQR) | 1.15 (1.03,1.27) | 1.14 (1.02,1.26) | 0.607 |
| Cl^-^ (mmol/L), median (IQR) | 103.00 (100.00,105.00) | 103.00 (99.00,105.00) | 0.266 |
| K^+^ (mmol/L), median (IQR) | 4.00 (3.50,4.80) | 4.00 (3.40,4.70) | 0.923 |
| Na^+^ (mmol/L), median (IQR) | 134.00 (129.00,138.00) | 133.00 (129.00,137.00) | 0.359 |
| Lactate (mmol/L), median (IQR) | 4.00 (1.80,8.40) | 3.60 (1.80,8.10) | 0.743 |
| PaCO_2_ (mmHg), median (IQR) | 40.80 (36.70,47.50) | 40.30 (35.00,47.30) | 0.192 |
| Total bilirubin (umol/L), median (IQR) | 12.30 (6.50,38.10) | 10.90 (5.40,31.20) | 0.069 |
| Triglyceride (mmol/L), median (IQR) | 0.96 (0.68,1.73) | 1.08 (0.65,1.64) | 0.782 |
| Total protein (g/L), median (IQR) | 55.00 (45.90,63.10) | 55.60 (47.30,64.70) | 0.454 |
| Hemoglobin (g/L), median (IQR) | 110.00 (92.00,125.00) | 109.00 (91.00,125.00) | 1.000 |
| Red blood cell (10^12^/L), median (IQR) | 3.87 (3.18,4.46) | 3.82 (3.11,4.52) | 0.871 |
| White blood cell (10^9^/L), median (IQR) | 11.55 (7.43,16.07) | 10.10 (6.40,14.93) | 0.083 |

**Table S9. Baseline characteristics of sodium bicarbonate treated and untreated children matched using propensity score matching method when 107 mmol/L≤chloride < 113 mmol/L.**

| **Variables** | **Without SB, n=447** | **With SB, n=447** | ***p*** |
| --- | --- | --- | --- |
| Gender, n(%) |  |  | 0.788 |
| Female | 200 (44.74) | 196 (43.85) |  |
| Male | 247 (55.26) | 251 (56.15) |  |
| Age, n(%) |  |  | 0.645 |
| ≤28 days | 77 (17.23) | 65 (14.54) |  |
| 28 days -1 year | 163 (36.47) | 161 (36.02) |  |
| 1-3 year | 96 (21.48) | 98 (21.92) |  |
| 3 years or above | 111 (24.83) | 123 (27.52) |  |
| Metabolic acidosis grade, n(%) |  |  |  |
| Mild | 390 (87.25) | 385 (86.13) | 0.515 |
| Moderate | 49 (10.96) | 57 (12.75) |  |
| Severe | 8 (1.79) | 5 (1.12) |  |
| Blood/immune diseases, n(%) | 8 (1.79) | 9 (2.01) | 0.807 |
| Circulation system disease,n (%) | 46 (10.29) | 43 (9.62) | 0.738 |
| Digestive system diseases, n(%) | 72 (16.11) | 67 (14.99) | 0.644 |
| Genitourinary disease, n(%) | 6 (1.34) | 5 (1.12) | 0.762 |
| Congenital diseases, n(%) | 171 (38.26) | 176 (39.37) | 0.731 |
| Injury or poisoning, n(%) | 21 (4.70) | 27 (6.04) | 0.373 |
| Sepsis, n(%) | 18 (4.03) | 17(3.80) | 0.863 |
| Pneumonia, n(%) | 37 (8.28) | 41 (9.17) | 0.635 |
| Meningoencephalitis, n(%) | 9 (2.01) | 12 (2.68) | 0.508 |
| Surgery, n(%) | 269 (60.18) | 275 (61.52) | 0.681 |
| pH, median (IQR) | 7.31 (7.27,7.33) | 7.31 (7.27,7.33) | 0.648 |
| Ca^2+^ (mmol/L), median (IQR) | 1.21 (1.13,1.28) | 1.21 (1.12,1.29) | 0.915 |
| Cl^-^ (mmol/L), median (IQR) | 110.00 (109.00,111.00) | 110.00 (109.00,111.00) | 0.702 |
| K^+^ (mmol/L), median (IQR) | 3.80 (3.30,4.30) | 3.80 (3.40,4.30) | 0.387 |
| Na^+^ (mmol/L), median (IQR) | 137.00 (135.00,140.00) | 137.00 (134.00,140.00) | 0.983 |
| Lactate (mmol/L), median (IQR) | 2.20 (1.20,4.00) | 2.10 (1.30,3.70) | 0.717 |
| PaCO_2_ (mmHg), median (IQR) | 40.40 (37.00,44.10) | 40.20 (36.90,44.70) | 0.886 |
| Total bilirubin (umol/L), median (IQR) | 8.20 (5.20, 26.50) | 8.80 (5.40, 23.40) | 0.684 |
| Triglyceride (mmol/L), median (IQR) | 1.05 (0.70, 1.51) | 1.07 (0.71, 1.53) | 0.487 |
| Total protein (g/L), median (IQR) | 61.60 (52.10, 68.30) | 60.90 (52.60, 68.00) | 0.678 |
| Hemoglobin (g/L), median (IQR) | 119.00 (105.00, 130.00) | 117.00(103.00, 131.00) | 0.394 |
| Red blood cell (10^12^/L), median (IQR) | 4.26 (3.62, 4.68) | 4.21 (3.56, 4.68) | 0.675 |
| White blood cell (10^9^/L), median (IQR) | 9.52 (7.01, 13.30) | 9.22 (6.82, 13.15) | 0.435 |

**Table S10. Baseline characteristics of sodium bicarbonate treated and untreated children matched using propensity score matching method when 113 mmol/L≤chloride.**

| **Variables** | **Without SB, n=438 (%)** | **With SB, n=438 (%)** | ***p*** |
| --- | --- | --- | --- |
| Gender, n(%) |  |  | 0.734 |
| Female | 196 (44.75) | 191 (43.61) |  |
| Male | 242 (55.25) | 247 (56.39) |  |
| Age, n(%) |  |  | 0.816 |
| ≤28 days | 72 (16.44) | 68 (15.53) |  |
| 28 days -1 year | 176 (40.18) | 166 (37.90) |  |
| 1-3 year | 108 (24.66) | 114 (26.03) |  |
| 3 years or above | 82 (18.72) | 90 (20.55) |  |
| Metabolic acidosis grade, n(%) |  |  |  |
| Mild | 373 (85.16) | 356 (81.28) | 0.136 |
| Moderate | 54 (12.33) | 74 (16.89) |  |
| Severe | 11 (2.51) | 8 (1.83) |  |
| Blood/immune diseases, n(%) | 6 (1.37) | 8 (1.83) | 0.590 |
| Circulation system disease, n(%) | 32 (7.31) | 31 (7.08) | 0.896 |
| Digestive system diseases, n(%) | 68 (15.53) | 70 (15.98) | 0.853 |
| Genitourinary disease, n(%) | 7 (1.60) | 8 (1.83) | 0.795 |
| Congenital diseases, n(%) | 199 (45.43) | 192 (43.84) | 0.634 |
| Injury or poisoning, n(%) | 25 (5.71) | 28 (6.39) | 0.671 |
| Sepsis, n(%) | 15 (3.42) | 18 (4.11) | 0.594 |
| Pneumonia, n(%) | 26 (5.94) | 26 (5.94) | 1.000 |
| Meningoencephalitis, n(%) | 10 (2.28) | 13 (2.97) | 0.526 |
| Surgery, n(%) | 290 (66.21) | 296 (67.58) | 0.667 |
| pH, median (IQR) | 7.31 (7.27,7.33) | 7.31 (7.27,7.33) | 0.150 |
| Ca^2+^ (mmol/L), median (IQR) | 1.21 (1.12,1.26) | 1.20 (1.12,1.27) | 0.925 |
| Cl^-^ (mmol/L), median (IQR) | 115.00 (114.00,117.00) | 115.00 (114.00,118.00) | 0.079 |
| K^+^ (mmol/L), median (IQR) | 3.60 (3.20,4.10) | 3.60 (3.20,4.10) | 0.459 |
| Na^+^ (mmol/L), median (IQR) | 139.00 (136.00,142.00) | 139.00 (136.00,143.00) | 0.829 |
| Lactate (mmol/L), median (IQR) | 1.65 (1.00,2.80) | 1.80 (1.10,3.10) | 0.180 |
| PaCO_2_ (mmHg), median (IQR) | 39.25 (35.40,43.40) | 39.15 (35.40,43.40) | 0.882 |
| Total bilirubin (umol/L), median (IQR) | 7.90 (5.10,22.10) | 7.90 (5.10,28.20) | 0.985 |
| Triglyceride (mmol/L), median (IQR) | 1.21 (0.78,1.75) | 1.14 (0.70,1.73) | 0.303 |
| Total protein (g/L), median (IQR) | 61.90 (53.40,67.80) | 61.60 (53.10,67.70) | 0.813 |
| Hemoglobin (g/L), median (IQR) | 118.00 (105.00,134.00) | 117.00 (105.00,131.00) | 0.536 |
| Red blood cell (10^12^/L), median (IQR) | 4.28 (3.74,4.77) | 4.34 (3.69,4.73) | 0.988 |
| White blood cell (10^9^/L), median (IQR) | 9.93 (7.30,12.61) | 9.35 (7.04,13.22) | 0.518 |
